# Global transcriptomic responses orchestrate difenoconazole resistance in *Penicillium* spp. causing blue mold of stored apple fruit

**DOI:** 10.1186/s12864-020-06987-z

**Published:** 2020-08-24

**Authors:** Franz J. Lichtner, Verneta L. Gaskins, Kerik D. Cox, Wayne M. Jurick

**Affiliations:** 1 Geisel School of Medicine, Dartmouth College, Hanover, NH 03755 USA; 2 U.S. Department of Agriculture-Agricultural Research Service, Beltsville, MD 20705 USA; 3 Plant Pathology and Plant-Microbe Biology Section, Cornell University, Geneva, NY 14456-0462 USA

**Keywords:** Transcriptomics, Azole fungicides, Blue mold, *Penicillium spp.*, Global gene networks, Active efflux pumps, Postharvest decay, Transcriptional regulators, Antimicrobial resistance

## Abstract

**Background:**

Blue mold is a globally important and economically impactful postharvest disease of apples caused by multiple *Penicillium* spp*.* There are currently four postharvest fungicides registered for blue mold control, and some isolates have developed resistance manifesting in decay on fungicide-treated fruit during storage. To date, mechanisms of fungicide resistance have not been explored in this fungus using a transcriptomic approach.

**Results:**

We have conducted a comparative transcriptomic study by exposing naturally-occurring difenoconazole (DIF) resistant (G10) and sensitive (P11) blue mold isolates to technical grade difenoconazole, an azole fungicide in the commercial postharvest product Academy (Syngenta Crop Protection, LLC). Dynamic changes in gene expression patterns were observed encompassing candidates involved in active efflux and transcriptional regulators between the resistant and sensitive isolates. Unlike other systems, 3 isoforms of cytochrome P450 monoxygenase (CYP51A-C) were discovered and expressed in both sensitive and resistant strains upon difenoconazole treatment. Active efflux pumps were coordinately regulated in the resistant isolate and were shown to mediate the global resistance response as their inhibition reversed the difenoconazole-resistant phenotype in vitro.

**Conclusions:**

Our data support the observation that global transcriptional changes modulate difenoconazole resistance in *Penicillium* spp. While the dogma of CYP51 overexpression is supported in the resistant isolate, our studies shed light on additional new mechanisms of difenoconazole resistance on a global scale in *Penicillium* spp. These new findings broaden our fundamental understanding of azole fungicide resistance in fungi, which has identified multiple genetic targets, that can be used for the detection, management, and abatement of difenoconazole-resistant blue mold isolates during long-term storage of apples.

## Background

*Penicillium* spp. are the most destructive postharvest fungal plant pathogens causing blue mold on stored fruits and vegetables worldwide. This group of fungi causes losses in high value crops like apple, pear, and quince fruit (pomes) and contributes to mycotoxin accumulation (patulin) in processed fruit products (e.g. apple sauce, butter, jams, juices). Control is achieved using multiple applications of postharvest fungicides and there is no host-based resistance to blue mold in commercial apple cultivars [1]. Since fungicides are relied upon and utilized as the main decay control method, it’s not surprising that antimicrobial resistant (AMR) strains are readily detected in the field and during storage [1–3]. Cultural controls (i.e. orchard sanitation, bin sterilization, proper cold chain management) are important components of integrated pest management and have been shown to reduce blue mold [4]. However, their implementation is limited and sporadically practiced in the industry and thus have not impacted the ubiquitous presence of fungal spores in the soil, air and water used in agricultural production systems. A comprehensive understanding of how fungicide resistance develops in *Penicillium* spp. is needed to develop rapid detection methods and strategies, alongside the synthesis of alternative controls with little to no risk of developing resistance to ensure maintenance of high-quality fruit during storage.

Azole resistance in both clinical and environmental samples has been attributed to mutations in ergosterol biosynthetic gene lanosterol 14α-demethylase (ERG11 in *Saccharomyces cerevisiae*) CYP51A, but recently, with more sampling and thorough sequencing, additional mutations are being associated with multi-azole resistance [5]. CYP51 conserved motifs required for ergosterol biosynthesis include: PFGXGRHRCXGEXFAY, PXHSXXR, AGQHTS, and AEEXYXXLTTPVFGKXVVYDCPNXXLMEQKKFVKXGL. They represent the heme binding domains which are also specific binding targets for azole fungicides [6] and are termed sterol regulatory element-binding proteins (SREBPs). It has been observed that the 1 kilobase (kb) region upstream of the start codon for CYP51A is important in the regulation or expression of the primary target for azoles [7, 8]. Findings from a paralogous system has shown that prolonged exposure to voriconazole increased transcript levels of a CYP51 gene in *A. fumigatus* [9]. *Penicillium digitatum* possess a 199 bp insertion and 126 bp tandem repeat transcriptional enhancer element with resistance to prochloraz [10, 11]. These genetic elements have been implicated in azole resistance in many filamentous fungi due to increased expression of the target gene by increasing promoter activity. Associated point mutations in the CYP51A gene (e.g. Y126F) have been shown recently to be associated with UV-generated *P. expansum* isolates with resistance to difenoconazole [12]. Mutation in the CYP51A encoded protein is hypothesized to be important for creating an insensitive target enzyme, in which the difenoconazole fungicide cannot bind and inhibit ergosterol biosynthesis.

Epigenetic regulation in human and plant pathogenic fungi is one a contributing factor of antifungal resistance [13]. There have been other hallmarks besides CYP51 transcript activation mediating azole resistance due to ABC transporter upregulation and MFS overexpression for active fungicide efflux, particularly in medically important pathogens like *Candidia albicans* in which fluconazole is prescribed to combat yeast infections in humans [14]. In fungal phytopathogens, regulation of active efflux genes was observed through an insertion in the MFS1 promoter, a 519-bp long terminal repeat (LTR), which led to multidrug-resistant strains of *Zymoseptoria tritici.* This insertion occurs in three different variations, which introduces different transcription factor binding sites, in two of the three CYP51 loci [15]. Multiple chromosome duplication events have been demonstrated for *Cryptococcus neoformans* that manifest in resistance when exposed to azole fungicides in clinical samples [16].

A recent study [1] provided the foundation for this transcriptomic investigation as the authors determined the effective concentration of difenoconazole to inhibit 50% mycelial growth (EC_50_) for 97 blue mold isolates that encompass multiple *Penicillium* spp. from many locations around the world*.* From this baseline difenoconazole-sensitive population, three isolates were able to grow on a discriminatory dose of 5 ppm difenoconazole, but only one isolate G10 did so consistently and rigorously [1]. Therefore, to understand novel mechanisms of azole resistance in *Penicillium* spp. infecting pome fruit, we investigated transcriptional changes between one difenoconazole-resistant (G10) and one difenoconazole sensitive (P11) isolate via a comparative transcriptomic approach. The objectives of the study were to: 1) determine differences in the gene expression profiles between resistant and sensitive isolates upon exposure to difenoconazole, 2) identify specific gene classes (MFS, ABC transporters, MDRs, and transcription factors) associated with the difenoconazole resistance phenotype and 3) put transcriptomic findings into biological context by challenging the G10 *Penicillium* spp. difenoconazole resistance mechanism using broad spectrum pump inhibitors. Results from our current investigation will be coupled with ongoing functional genetic and omics-based studies in the fungus to target and monitor suites of genes involved in fungicide resistance to design rapid genetic-based detection methods, formulate new chemistries less likely to develop resistance, and synthesize additional compounds to augment chemically-based controls to mitigate fungicide-resistant blue mold isolates.

## Results

### *Penicillium* spp. characterization and pathogenic fitness in apple

**Fig. 1:**
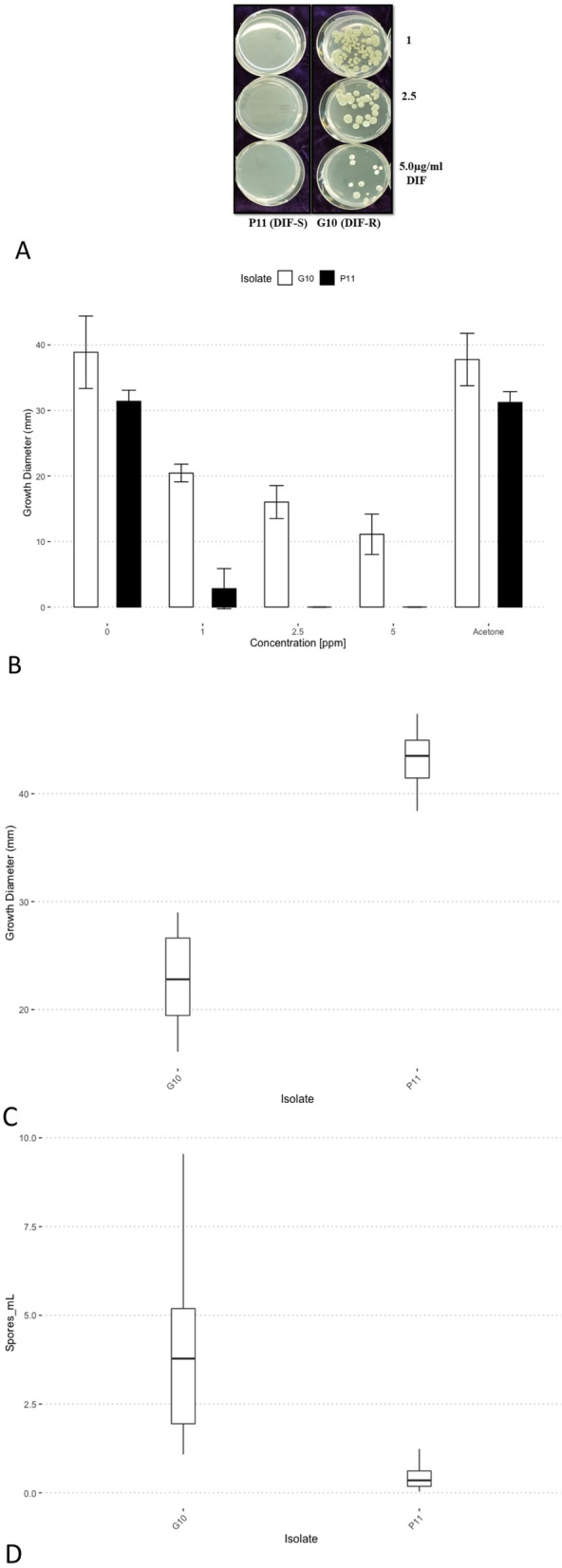
Growth of fungicide resistant (G10) and sensitive (P11) *Penicillium* spp. on 5 μg/ml difenoconazole (DIF), growth rate in culture, and conidial production in vitro*,* and virulence in apple fruit*.* **a** *Penicillium* spp. isolates P11 and G10 growing on 1, 2.5 and 5 μg/ml difenoconazole amended Potato Dextrose Agar (PDA), **b** Colony diameters of P11 and G10 on 1, 2.5 and 5 μg/ml difenoconazole-amended and unamended PDA, **c** Lesion size in apple fruit inoculated with G10 and P11 7 days post inoculation. **d** Conidial production of G10 and P11 on PDA in vitro

Differences between difenoconazole-resistant *P. crustosum* G10 and difenoconazole-sensitive *P. expansum* P11 were investigated on difenoconazole-amended media (Fig. 1a). Growth on different concentrations of difenoconazole amended potato dextrose agar (PDA) resulted in colony diameters of 20.5 mm [1 ppm], 16 mm [2.5 ppm], and 11 mm [5 ppm] for G10 while P11 only grew 2 mm [1 ppm] (Fig. 1b). The mean colony diameter on diagnostic media (MEA, CYA and YES) for the resistant isolate G10 was 30.96 mm on (MEA) while P11 was 27.95 mm. However, on yeast extract with supplements (YES), G10 and P11 had colony diameters of 25.4 mm and 26.8 mm 25 °C 7 days post inoculation (dpi) (Supplemental Figs. S1 and S2). Mean lesion size for both isolates inoculated onto wounded ‘Golden Delicious’ apple fruit were 22.93 mm for G10 and 43.19 mm for P11 at 25 °C after 7 days and were significantly different from one another (*p* < 2.2e-16) (Fig. 1c). Conidial production for G10 was 2–5 × 10^6^ spores/mL and P11 produced less than 1 × 10^6^ spores/mL obtained from 7-day old Petri plates containing PDA which was significantly different from each other (*p* < 5.7e-09) (Fig. 1d). To determine if difenoconazole resistance was inherent to other *P. crustosum* species, we tested 3 other *P. crustosum* isolates (R1, R14, and G20) for growth on 5 μg/ml difenoconazole from a baseline sensitive *Penicillium* spp. population previously described [1]. None of these isolates grew on the discriminatory dose and were found to be sensitive (data not shown).

### Phylogenomic analysis of *Penicillium* spp.

The phylogenetic relationship of the isolates examined in this study were compared to other relevant *Penicillium* species. The *Penicillium* spp. genomes sequenced, assembled and analyzed in this study (e.g. G10 and P11) revealed high quality assemblies as indicated by Basic Universal Single Copy Orthologs (BUSCO) completeness percentage (Supplemental Table S1). Phylogenetic analysis was accomplished using 14 closely related *Penicillium* spp. genomes, with 3117 paralogous genes, which resulted in a unique taxonomic grouping. All the previously published *P. expansum* genomes plus *P. expansum* P11 grouped together with 100% boot strap support (Supplemental Fig. S3). *Penicillium crustosum* isolate G10 (difenoconazole resistant) grouped more closely to *P. solitum* IBT 29525, and *P. polonicum* IBT 4502 [17].

### Phylogenetic analysis of CYP51 isoforms and their differential gene expression

**Fig. 2:**
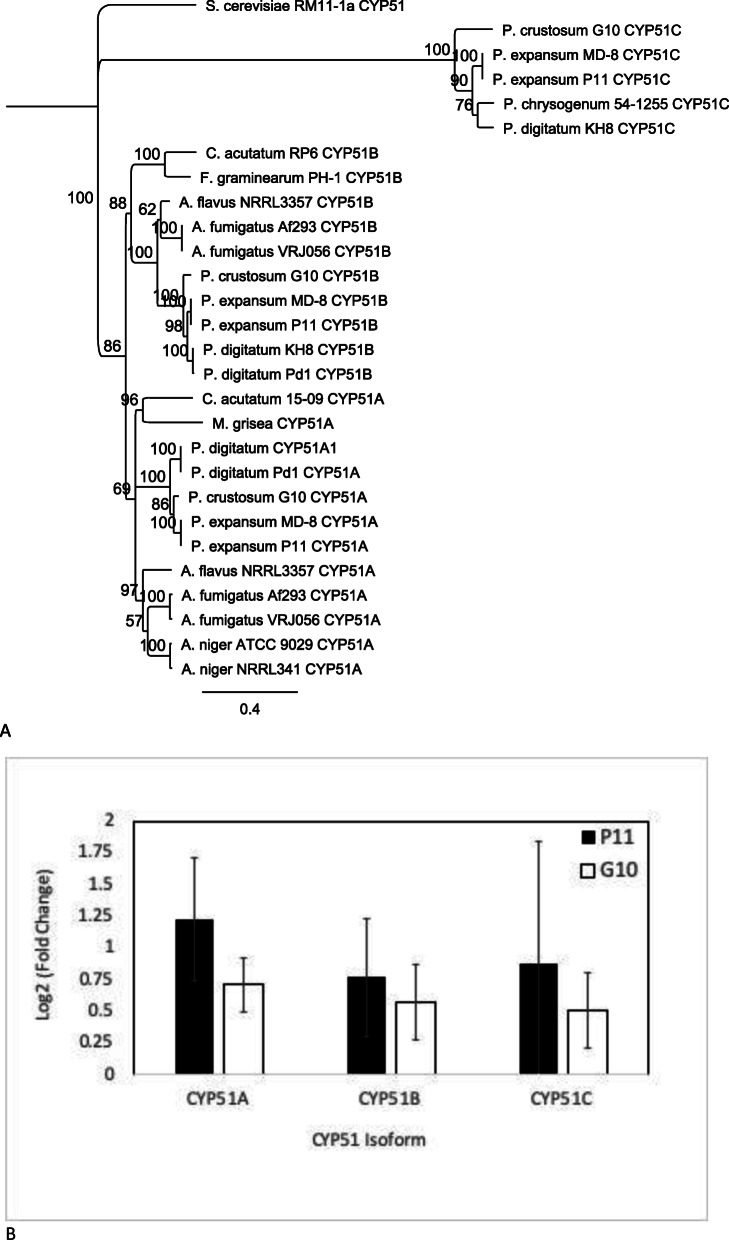
Phylogeny and differential expression of Cytochrome P450 loci in *Penicillium* spp. **a** Phylogenetic analysis of CYP51 genes from *Penicillium* species, **b** Quantitative Real time Polymerase Chain Reaction (qRT-PCR) gene expression of CYP51 A, B, and C genes

Three unique clades with high bootstrap support (100% with 100 bootstraps) were observed, when three CYP 51A, B, and C isoforms from G10 and P11, were analyzed via phylogenetic analysis (Fig. 2a). Twenty-two previously published CYP51A, B, and C coding sequences plus 6 from this study were included that encompassed 11 different genera: *P. expansum*, *P. digitatum*, *P. crustosum*, *P. chrysogenum*, *Saccharomyces cerevisiae*, *Colletotrichum acutatum*, *Fusarium graminearum*, *Aspergillus flavus*, *A. fumigatus*, *A. niger*, and *Magnaporthe grisea*. The CYP51C sequences of *P. expansum*, *P. chrysogenum*, *P. digitatum* and *P. crustosum* all grouped separately from the CYP51A and CYP51B from the others listed above. CYP51A and CYP51B each had three internal clades where *Aspergillus*, *Penicillium* and other genera separated with 100 to 96% bootstrap support. Quantitative real time expression data, presented as log_2_(Fold Change) (Log_2_FC) of difenoconazole normalized expression divided by acetone control, of the three CYP51 isoforms from both isolates showed that each gene was overexpressed in P11 and G10 when exposed to difenoconazole (Fig. 2b). Briefly, CYP51A Log_2_FC was 0.71 (+/− 0.21) for G10 and 1.22 (+/− 0.48) for P11, G10 CYP51B Log_2_FC was 0.57 (+/− 0.31) and P11 Log_2_FC was 0.77 (+/− 0.47), and the CYP51C Log_2_FC was 0.51 (+/− 0.29) for G10 and 0.88 (+/− 0.95) for P11.

The CYP51A cDNA from P11 was 100% identical to PEX2_004640 from *P. expansum* MD8 while CYP51A from G10 was 92.3% similar. G10 and P11 CYP51A polypeptide sequences showed single amino acid differences in the ion channel and GET4/5 biding domain, but were identical in the heme binding, SRS1 and SRS4 domains (data not shown). The coding sequences of the difenoconazole-responsive CYP51B gene from P11 and G10 were analyzed and found to be devoid of the Y126F mutation associated with resistance to difenoconazole [18] and contained no mutations in the highly conserved heme binding domain located near the C-terminus of the polypeptide (data not shown). Approximately 1 kb sequence upstream of the CYP51A, B and C putative start sites were examined for G10 and P11 and did not reveal insertions or tandem repeats previously identified in other fungal systems with azole fungicide resistance (data not shown).

### RNA sequencing and comparative transcriptomics

**Fig. 3:**
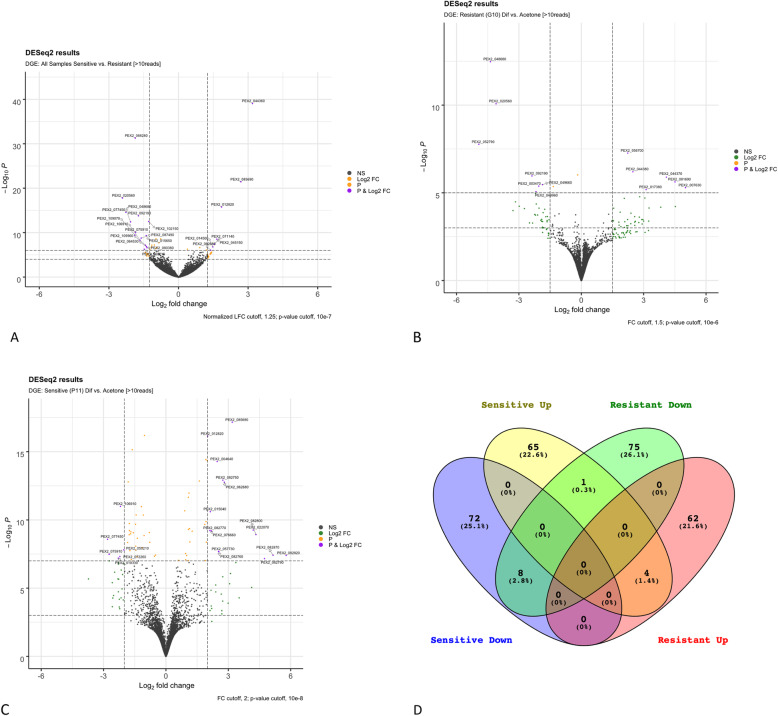
Plots showing RNA-seq data for difenoconazole resistant (G10) and sensitive (P11) *Penicillium* spp. **a** genes expressed amongst all treatments in both resistant (G10) and sensitive (P11) isolates (Log2Fold > 1.25 DGE +/−), **b** difenoconazole-responsive transcripts in resistant G10 (treatment/control) and **c** difenoconazole-responsive genes in sensitive P11 (treatment/control). **d** Venn diagram showing the top 150 genes up and down regulated in sensitive and resistant isolates in response to difenoconazole treatment

Transcriptome analysis resulted in an average of 34,056.125 transcripts per sample, the mean number of transcripts > 1000 bp was 13,795.875, average number of GeneMarkS-T genes was 20,252.8, and average database coverage of 74.75% determined with rnaQUAST [19]. The mean number of unaligned transcripts was 4445.68 and the average number of mismatches per transcript was 32.2. Significantly differentially expressed genes were analyzed as treatment over control which allows observation of expression patterns of each individual isolate during the difenoconazole treatment. Gene expression of select loci was plotted across all treatments and strains, for resistant G10 only and sensitive P11 based on *p*-value cut off and log2(FoldChange) (Fig. 3a-c). The top 150 significantly differentially expressed genes for (treatment over control) analysis were identified to create a Venn Diagram showing the associated up and down regulated genes for each category and where they overlap (Fig. 3d).

The overall distribution of differentially expressed transcripts amongst all treatments was skewed toward those that were upregulated. There were 27 genes significantly upregulated and 12 down regulated (Fig. 3a). The most significantly upregulated was PEX2_032540 a pepsin like aspartic protease. Another significantly upregulated gene was PEX2_004930 encoding a ABC transporter, and most significantly down regulated was a pectinesterase (PEX2_040830). Normal distribution of transcripts was observed for G10 difenoconazole-responsive genes as 7 loci were down regulated and 6 upregulated that encoded an array of CAZymes, transporters, and cellular detoxification (Fig. 3b). For example, Cytochrome P450 Type 2 was the highest upregulated transcript and a hypothetical protein was the most downregulated. Other loci that were significantly increased included glutathione-S-transferase, a glycoside hydrolase and an ATPase. Significantly downregulated genes included MFS, FAD oxidase, peptide transport, and two containing Domains of Unknown Function (DUF). For the sensitive P11 strain, data was slightly skewed toward upregulated responses that encompassed 15 difenoconazole-responsive genes and 6 downregulated (Fig. 3c). The highest upregulated transcript encoded a glucose dehydrogenase and the greatest downregulated transcript encoded a peptidase. In order to observe similarities, differences and uniquely expressed genes, the top 150 most significantly differentially expressed genes were analyzed using a Venn diagram (Fig. 3d). There were 8 genes downregulated in both sensitive and resistant isolates in response to difenoconazole (Fig. 3d). Four genes were upregulated in both resistant and sensitive isolates, and only one gene was upregulated in the sensitive isolate but downregulated in the resistant isolate, PEX2_077570, a cytochrome P450.

**Fig. 4:**
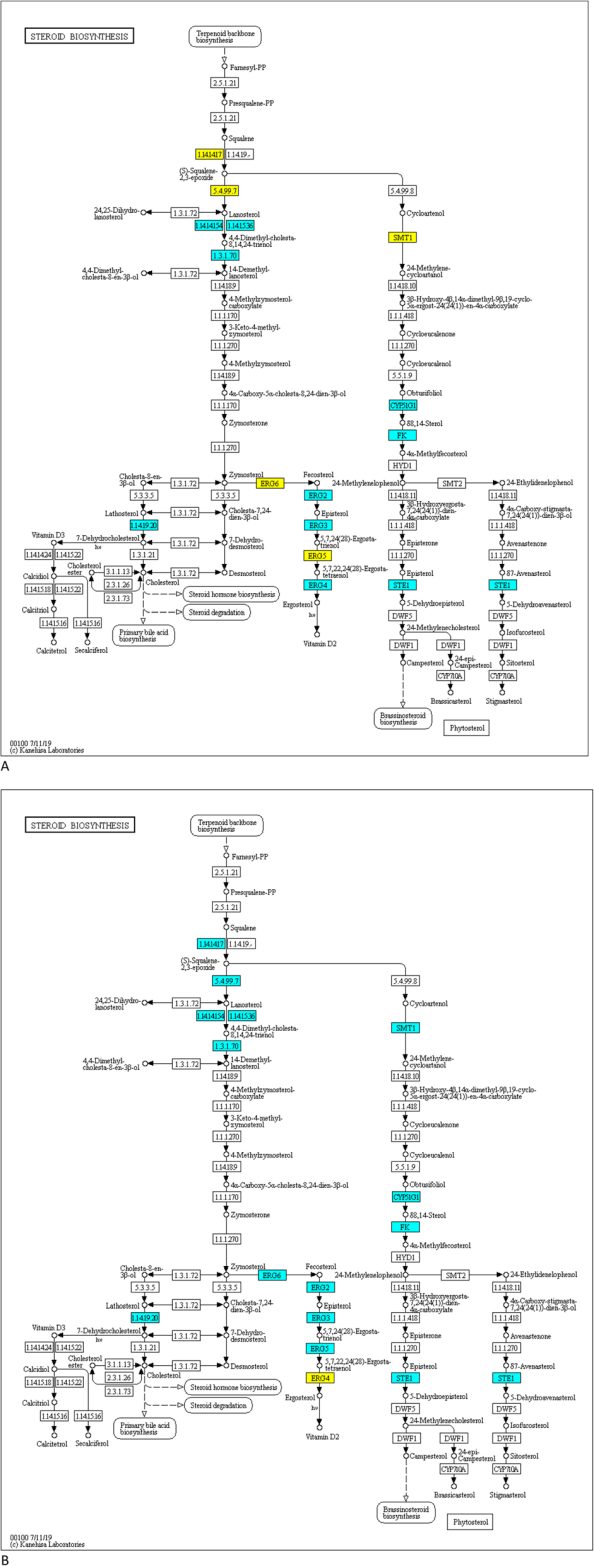
KEGG map of ergosterol pathway genes differentially regulated, upregulated in blue and downregulated in yellow, in *Penicillium* species **a** G10 and **b** P11 isolates after exposure to 5 μg/ml difenoconazole. Copyright permission was granted for use and modification of: M00101 Cholesterol biosynthesis, squalene 2,3-epoxide = > cholesterol. M00102 Ergocalciferol biosynthesis, squalene 2,3-epoxide = > ergosterol/ergocalciferol. M00917 Phytosterol biosynthesis, squalene 2,3-epoxide = > campesterol/sitosterol. M00103 Cholecalciferol biosynthesis

**Fig. 5:**
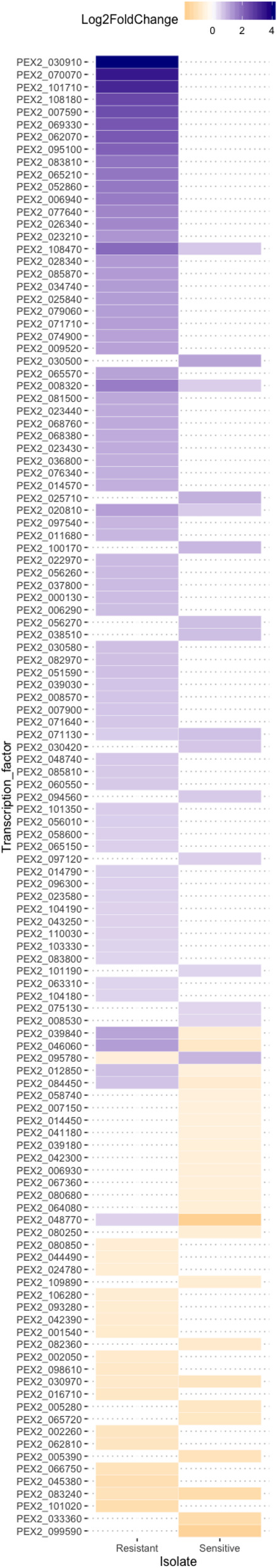
Heat map of Zn-Cys transcription factors expressed in G10 (Resistant) and P11 (sensitive) isolates

**Fig. 6:**
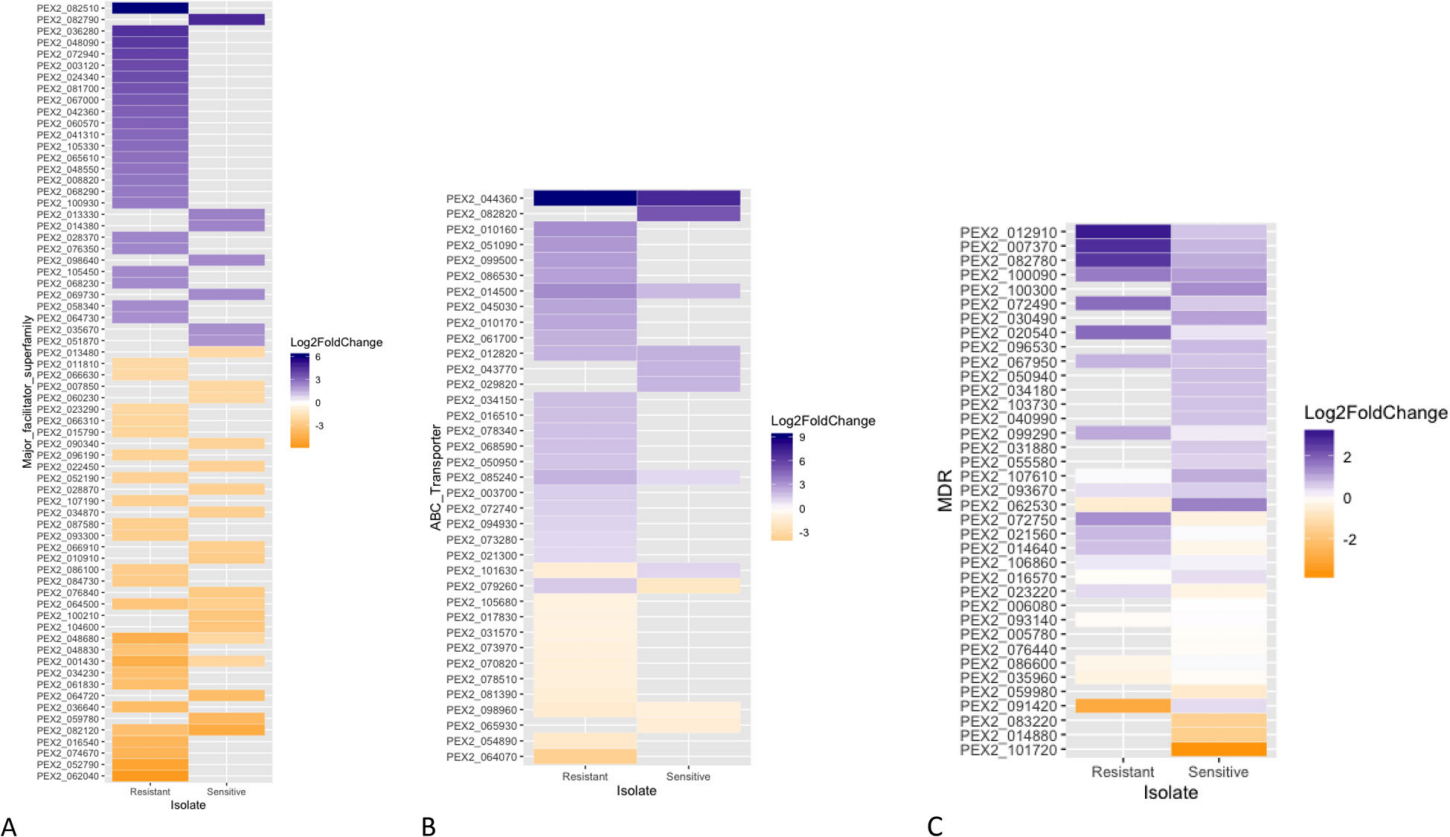
Heat maps showing targeted comparative expression analysis for specific classes of pumps and transcription factors in G10 and P11 **a** Multi-Function Substrate, **b** ATP Binding Cassette, **c** Multi Drug Resistance

**Fig. 7:**
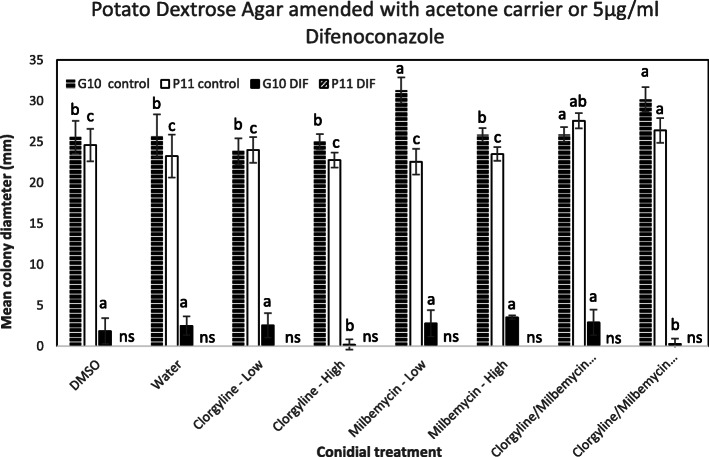
Dose dependent inhibition of a difenoconazole (DIF) resistant (G10) *Penicillium* spp. isolate using conidia treated with broad spectrum active efflux pump inhibitors and plated on Potato Dextrose Agar amended with acetone (control) or 5 μg/ml difenoconazole. Growth of *Penicillium* spp. G10 and P11 conidial suspensions growing on difenoconazole-amended and acetone-amended Potato Dextrose Agar with DMSO carrier, water, clorgyline (25 or 250 μm), milbemycin oxime (10 or 100 μm), clorgyline and milbemycin oxime (25 and 10 μm), and clorgyline and milbemycin oxime (250 and 100 μm)

The resistant isolate (G10) expressed the ATPase and heat shock protein (HSP) gene classes more and were often not expressed in the sensitive (P11) isolate. For ABC-transporters the CDR ABC transporter PEX2_044360 was significantly over expressed in the resistant isolate compared to the sensitive. The alcohol dehydrogenase super family zinc type PEX2_012910 MDR was upregulated in the resistant compared to sensitive (Table 1). Genes exhibiting the opposite expression patterns between G10 and P11 for ERG6, 5, and 4. ERG6 and 5 genes are downregulated in G10 but up regulated in P11, yet the ERG4 gene is up regulated in G10 but downregulated in P11 in the presence of difenoconazole (Fig. 4). We observed TF expression in G10 that is remarkably different than that of P11 with PEX2_030910 Zn (2) Cys (6) (pfam 00172) being Log2FC > 4 upregulated when exposed to difenoconazole (Fig. 5) The resistant (G10) isolate more highly expressed multiple classes of Multi-Function Substrate (MFS) (PEX2_082510 and PEX2_36280), ATP-Binding Cassette (ABC) (PEX2_044360), and Multi-Drug Resistant (MDR) (PEX2_012910) related genes compared to the P11 sensitive isolate (Fig. 6a-c). The involvement of active efflux pumps was further investigated using broad spectrum pharmacological inhibitors. Clorgyline, but not milbemycin oxime reversed difenoconazole resistance in G10 in a dose-dependent fashion alone and in combination with milbemycin oxime (Fig. 7).

**Table 1:** The top 5 most differentially expressed genes that are downregulated in difenoconazole-resistant G10 and upregulated in difenoconazole-sensitive P11. The five most upregulated genes in G10 but downregulated in P11. Also, the top 5 most statistically significant transcripts amongst the transcriptome data set are shown

Gene	Function	baseMean	Log Fold Change	Resistant (G10)	Sensitive (P11)	Adjusted ***P*** value
PEX2_103120	**Aldo/keto reductase**	280.5	10.64	DOWN	UP	6.10092E-22
PEX2_082770	**Glucose-methanol-choline oxidoreductase, C-terminal**	772.75	9.83	DOWN	UP	2.04811E-28
PEX2_090570	**hypothetical protein**	151.78	9.76	DOWN	UP	3.27597E-19
PEX2_063220	**hypothetical protein**	530.25	9.67	DOWN	UP	7.29109E-28
PEX2_087000	**HAD-superfamily hydrolase, subfamily IA, variant 3**	314.9	9.43	DOWN	UP	6.17432E-21
PEX2_038660	**hypothetical protein**	231.12	−11.5	UP	DOWN	4.68733E-24
PEX2_024490	**NAD-dependent epimerase/dehydratase**	12,942.41	−10.92	UP	DOWN	3.5552E-45
PEX2_080600	**AMP-dependent synthetase/ligase**	2219.3	−10.42	UP	DOWN	1.12256E-43
PEX2_089800	**hypothetical protein**	209.88	−10.06	UP	DOWN	2.02213E-26
PEX2_089790	**Sterile alpha motif, type 2**	79.93	−9.95	UP	DOWN	1.93409E-18
PEX2_014150	**hypothetical protein**	1416.75	−7.9	UP	DOWN	9.8643E-129
PEX2_067310	**Bicupin, oxalate decarboxylase/oxidase**	4486.4	6.61	DOWN	UP	1.08E-128
PEX2_013820	**Short-chain dehydrogenase/reductase SDR**	460.03	−6.8	UP	DOWN	2.24942E-93
PEX2_080200	**Major facilitator superfamily domain, general substrate transporter**	2103.93	−7.25	UP	DOWN	1.34062E-88
PEX2_035400	**Inositol monophosphatase**	2368.63	− 5.4	UP	DOWN	4.10718E-81

**Fig. 8:**
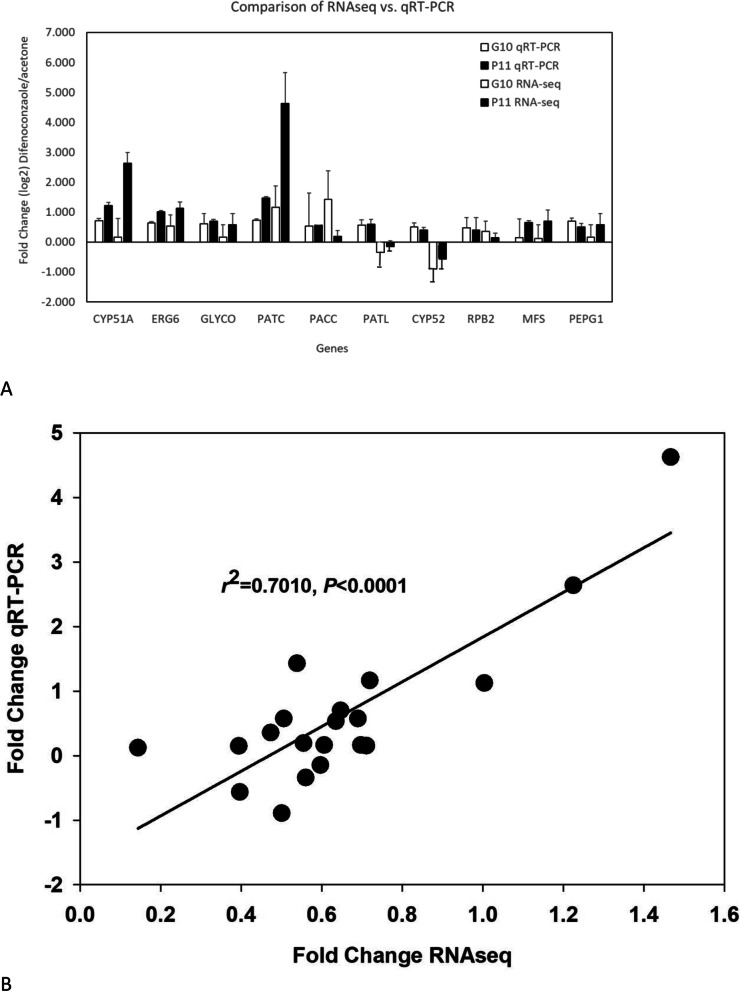
**a** Validation of differential gene expression in 9 different loci in G10 and P11 using quantitative reverse transcription polymerase chain reaction. **b** Regression analysis of fold change for select loci determined by qRT-PCR or RNAseq

Validation of differential gene expression data using qRT-PCR were generally in agreement with our bioinformatic analysis of RNA-seq data (Fig. 8a). Exceptions include PatL and CYP52 genes that both exhibited negative log_2_FC via RNA-seq but positive log_2_FC change in qRT-PCR. The other 8 genes tested via qRT-PCR showed similar trends and were consistent with bioinformatic findings from RNA-seq expression data. Regression analysis exhibited an *r*^2^ value of 0.701with *P <* 0.001 (Fig. 8b).

## Discussion

### Global transcriptomic changes occur in response to difenoconazole treatment

The aim of this study was to compare differential gene expression patterns in resistant vs. sensitive *Penicillium* spp. after exposure to a discriminatory dose of difenoconazole. The investigation was implemented to gain mechanistic insights into how *Penicillium* spp. develop resistance to postharvest fungicide treatment. Both sequences and morphological observations showed that one isolate (G10) is *P. crustosum* while the other (P11) is *P. expansum*. To date, there are no *P. crustosum* genomes available in Genbank or other online genomic resources to include in our analysis. Hence, our well annotated genome is the first *P. crustosum* isolate to be sequenced, annotated and released for public usage. Mechanisms of resistance to antifungals have been well described and characterized over the last 30 years in plant, animal and human pathogens [20]. However, our study highlights new mechanism(s) of resistance in *Penicillium* spp. and provides supporting biological data on the central resistance theme that involves global transcriptional regulation of active efflux pumps and suites of a specific class of transcription factor.

### CYP51 expression and ergosterol pathway regulation

It was hypothesized that CYP51A overexpression was a component of difenoconazole resistance mechanism which we observed for both sensitive and resistant isolates. Our bioinformatic analyses and expression data reveal that both G10 and P11 have three distinct CYP51 isoforms that are upregulated during difenoconazole treatment. While this has not been shown for sensitive isolates in the literature, it is plausible that the fungus is compensating and producing additional transcripts to flood the synthesis of CYP51 proteins to maintain sterol biosynthesis in the presence of difenoconazole. Additional studies of ergosterol and the ERG pathway intermediates using a metabolomic approach should be conducted by analyzing flux through the pathway in both sensitive and resistant isolates to help answer the biological basis of the observed increase in ERG11 transcription. Additionally, gene expression of the entire ergosterol biosynthetic pathway in both isolates lends itself to possible transcription factor (TF) redundancy and or alternative regulation to facilitate sterol biosynthesis in G10 but not in P11 in the presence of difenoconazole. In other systems, a *P. digitatum* sterol regulatory element-binding protein has been implicated in prochloraz resistance, acting as a transcription factor, as it is associated with virulence and regulating CYP51 gene expression [12]. Hence, it is possible that we have a similar situation, but will require further investigation at both the genetic and biochemical levels using targeted gene deletion of these loci in G10 and P11.

### Active efflux is a main contributor of difenoconazole resistance in *Penicillium* spp.

We suspected that additional mechanisms were in place in which resistance was more complicated than previously reported that involved CYP51a overexpression and associated point mutations (E.g. Y126F) in *P. expansum* [18]. Through comparative transcriptomic analysis, we observed increases in broad classes of genes, in response to difenoconazole exposure. Unlike resistance in other *Penicillium* spp. our data implicate multiple, coordinated mechanisms of difenoconazole resistance. Though not unique in filamentous fungi, active efflux has not been demonstrated in blue mold species, as a resistance mechanism to difenoconazole. For example, energy dependent active efflux, a component of resistance shown in isolate G10, is evident in the CDR1 (*Candida* drug resistant) ortholog PEX2_044360 ABC transporter. This locus represents a well-studied active efflux pump annotated in the *Candida albicans* genome, resides within in the pleiotropic drug resistance (PDR) class [21], and has been implicated in antifungal resistance but not in *Penicillium* spp. The over expression of this specific gene in the resistant G10 transcriptome indicates the potential for azole efflux leading to reduced fungicide accumulation in the cell [22]. Further evidence using chemical inhibition of active efflux pumps reversed the observed resistance phenotype in G10 in the presence of difenoconazole in a dose-dependent fashion but did not negatively impact fungal growth when compared to controls absent of difenoconazole. However quantitative studies to assay active pumping, difenoconazole efflux and differential levels of difenoconazole in G10 have yet to be conducted.

Active efflux has been demonstrated for other genera of plant and human fungal pathogens. *Zymoseptoria tritici*, an important plant pathogen of wheat, has been shown to develop resistance to many foliar-applied fungicides (e.g. azoles, succinate dehydrogenase inhibitors, multisite materials like chlorothalonil) through three genomic modifications involved in efflux [15]. These modifications include repetitive element insertions in the *MFS1* promoter which provides the basis for efflux gene overexpression. However, we did not observe this phenomenon in our resistant G10 isolate. Though the exact genetic mechanism of active efflux overexpression is unclear in *Penicillium* spp., it is evident that pumps are involved in G10 which are significantly different from those expressed in the sensitive isolate. Though we saw no evidence of insertions in repetitive elements upstream of the over expressed efflux genes, there may be other motifs and transcriptional regulators that are likely regulating their overexpression as evidenced by vast differences in zinc cys-type transcription factors.

*Candida albicans* implements active efflux through multiple overexpressed genes including CDR1 and MFS1 during triazole and fluconazole exposure [23]. Though these are two different classes of transporters (PDR and the DHA12) the PDR has the least amount of homology in yeasts and filamentous fungi, suggesting that this family of transporters undergoes adaptive evolution from environmental pressures in a species-specific manner [24]. We observed an increase in transporter overexpression in genes involved in active efflux, yet when broad spectrum pumps were inhibited, fungal growth still occurs on difenoconazole, implying that this mechanism is not solely responsible for resistance. However, definitive evidence will be obtained via targeted deletion of transcription factors responsible for pump expression and or silencing of multiple pump families using RNAi.

## Conclusions

As antimicrobial resistance continues to negatively impact agricultural and human health arenas, there needs to be in depth knowledge of the topic to enable efficient and reliable detection of and alternative control(s) for fungal pathogens. We demonstrated for the first time that coordinated, global transcriptomic changes mediate azole fungicide resistance in *Penicillium* spp. Results from this study have shown that both G10 and P11 have 3 CYP51 loci that are activated, which is unlike other fungal systems where CYP51A was overexpressed in response to difenoconazole. Additionally, multiple classes of active efflux pumps are upregulated in the resistant isolate, which were compromised by a broad-spectrum pump inhibitor. Differential expression of transcription factors was also observed between resistant and sensitive isolates, which further emphasize expression changes observed in multiple gene classes. These research findings move the current fundamental knowledge base forward concerning detailed molecular mechanisms of azole-mediated fungicide resistance in *Penicillium* spp. Continued annotation and refinement of fungal genomic resources and collaborative efforts, across multiple research disciplines in proteomics and metabolomics, will ultimately be translated to aid in limiting product losses and reduced productivity due to antimicrobial resistance. We aim to further explore upstream regulators of active efflux pumps, via functional analysis coupled with omics-based studies in our system, to dissect upstream regulation of the difenoconazole resistance mechanism. Our study is the first to identify multiple genetic targets that will propel future design of rapid detection tools, fungicide resistance management strategies, and tactics to abate difenoconazole-resistant blue mold isolates from developing during storage.

## Methods

### Morphological analysis of *Penicillium* spp. isolates

*Penicillium* spp. isolates were selected based on their sensitivities to difenoconazole which was previously determined for an unexposed blue mold population [1]. Two additional isolates were included for morphological comparison which were obtained from the USDA-ARS NRRL collection as reference strains including *P. expansum* NRRL 976 (type strain) and *P. crustosum* NRRL 968 (CBS 483.75). Cultural morphology (Supplemental Figure S1) and mycelial growth rates (Supplemental Figure S2) on three diagnostic media were conducted on Czapek Yeast Extract Agar (CYA), Malt Extract Agar (MEA) and Yeast Extract Sucrose (YES) at three temperatures as described [25].

### Virulence assessment in apple and conidial production

Virulence in apple fruit was evaluated using ten ‘Golden Delicious’ fruits which were first surface sanitized by washing with soap and water, rinsed clean, and sprayed with 70% ethanol and wiped dry with sterile paper towels. Fruit were then wounded with a 3 mm sanitized wounding tool that mimics a stem puncture. Ten μl of a 1 × 10^5^ conidia/ml suspension was prepared in filter sterilized Tween 20 treated water which was used to inoculate each wound. Control samples were wounded and inoculated with sterile Tween 20 treated water alone. After 30 min, apples were placed on standard cardboard fruit trays in industrial 80-count apple boxes for storage. Samples were incubated for 7 days post inoculation at 25 °C in the dark. At the end of the incubation period, the diameter of lesions on the fruits were measured with a digital hand-held micrometer. The experiment was conducted two times. Twenty apple fruit were used for the inoculation studies to determine the virulence of each isolate for each experiment. Apple fruit were obtained from the Penn State Fruit Research and Extension Center located in Biglerville, Pennsylvania at commercial harvest maturity with mean starch score of 3. To determine the isolates capacity to produce conidia, three 5 mm plugs of 7-day old P11 and G10 cultures were vortexed for 5 s in 1 ml Tween 20 treated water to create a spore suspension. Samples were taken from 3 separate Potato Dextrose Agar Petri dishes. Spores were quantified on a standard hemocytometer using a compound light microscope. The experiment was performed three times. Welch two sample t-test was used to statistically test differences in lesion size in apple and conidial production in vitro.

### CYP51 cloning, sequencing and isoform analysis

Gene specific primers were designed and used to amplify full coding regions from CYP51A from G10 and P11. Conventional PCR was implemented with standard cycling parameters that were specified per the manufacturer’s instructions with Q5 high fidelity polymerase (NEB, USA). Primer sequences can be found in supplemental file S5 and PCR products were cloned using TOPO blunt PCR cloning kit (Invitrogen, USA). Clones were screened using PCR and sequenced using M13F and M13R primers via Sanger Sequencing at Eurofins Inc. Sequences were assembled using online bioinformatic tools (Clustal Omega) and 2X consensus were generated and compared to genomic sequences using BLAST. Approximately 1 kb upstream region of CYP51A was generated, cloned and sequenced in the same manner. However, for CYP51B and C in silico sequences were obtained from transcriptome and genome data to generate these sequences. Online genomic comparison tools (NSITE program) and the eukaryotic promoter predictor (Berkeley Drosophila Genome) were used to analyze the promoter/upstream regions of CYP51A and B for transposons, repetitive elements and other hallmarks of mobile genetic elements. Multiple amino acid sequence alignments were generated using Clustal Omega for CYP51B for G10 and P11 along with online tools (ScanProsite) to search for conserved regions of biochemical function and motifs.

### Difenoconazole treatment and RNA isolation

Potato Dextrose Broth liquid cultures (50 ml) were inoculated with 100 μl of 1 × 10^^4^ conidia/ml from G10 or P11 isolate were grown for 3 days with gentle shaking (150 rpm) at 25 °C in a temperature-controlled incubator. Four cultures for each isolate (G10 or P11) were grown and there were 2 treatments: acetone (carrier control) or 5 μg/ml difenoconazole dissolved in acetone. One day (24 h) after addition of acetone or difenoconazole, cultures were harvested, and mycelial mats were lyophilized. Total RNA was isolated from approximately 1500 mg of tissue and were homogenized with a bead beater and the Trizol method according to the manufacturer’s instructions [26]. RNA was resuspended in DEPC-treated sterile water, and sample quality was assessed with gel electrophoresis on a 1% agarose gel at 75 V for 35 min, Nanodrop spectrophotometer (Thermofisher Scientific, Wilmington, DE) 260/280 and 260/230 ratio and Agilent 2100 Bioanalyzer for RNA integrity number (RIN) at BGI (Hong Kong, Peoples Republic of China). RNA sequencing performed on Illumina Hiseq 2500 paired end 2 × 150 bp reads.

### Whole genome sequencing, bioinformatic analyses and phylogenomic tree construction

Fungal mycelia from liquid shake cultures were frozen in liquid nitrogen, lyophilized and stored at − 80 °C. Lyophilized mycelia were used to isolate high quality, intact fungal genomic DNA according to Yelton et al. [27]. Genomic DNA was obtained, quantified via agarose gel electrophoresis using uncut lamba standards, and via nanodrop spectrophotometer with A260nm. Genomic DNA was sent to Cornell University, Institute of Biotechnology for quality control check and whole genome sequencing was accomplished using the Illumina MiSeq v2 500 bp platform (250 bp paired end reads, insert size of 500 bp). Assembly and annotation of genomes was conducted with shovill (https://github.com/tseemann/shovill) via a minimum contig size of 500 and funannotate (https://github.com/nextgenusfs/funannotate) with Augustus training using the *Penicillium expansum* R19 genome and other defaults.

The FASTQC analysis was performed to check the quality of the RNA-seq reads [28]. De novo assembly of the transcriptomes was performed under default settings with Trinity on Galaxy (usegalaxy.org) which included the flag for read trimming. The completeness and statistics of de novo assemblies was confirmed with rnaQUAST (Supplemental Table S1; additional file 6 Table S2) and final quantification of transcripts with Salmon [29]. Prefiltering of low count transcripts (≤10) before running DESeq2 including use of apeglm for LFC shrinkage [30]. Differential gene expression analysis was performed with DESeq2 in R [31] utilizing the GenomicFeatures, tximport and tximportData packages along with ggplot2, EnhancedVolcano and Venny 2.1 for visualization [32]. Mapping steroid biosynthesis genes to their cognate biochemical pathway was conducted via KEGG pathways reconstruction method using the log_2_FC from the RNA-seq differential gene expression from treatment over control for G10 and P11 (Fig. 4a and b) [33].

Genomes used to conduct the phylogenomic analysis include the following: *Penicillium expansum* (GCA_000769745.1) [34], *P. expansum* (GCA_004302965.1) [35], *P. expansum* P11 and *P. crustosum* G10 annotated genomes (unpublished). Published genomes incorporated in the analysis include *Aspergillus fumigatus* (GCA_000002655.1), *P. chrysogenum* (GCA_000710275.1), *P. polonicum* (GCA_002072265.1), *P. digitatum* (GCA_001307865.1), *P. italicum* (GCA_002116305.1), *P. roqueforti* (GCA_000737485.2), *P. expansum* (GCA_000769735.1), *P. solitum* (GCA_002072235.1), *P. verrucosum* (GCA_000970515.2), *P. paneum* (GCA_000577715.1), and *P. carneum* (GCA_000577495.1). Phylogenomic trees were constructed via BUSCO outputs, both nucleotide and amino acids, from run_BUSCO.py followed by the implementation of mafft [36] for alignment of proteins and nucleotides and raxml [37, 38] for tree building via the python script BuscoOrthoPhylo.py (https://github.com/PlantDr430/BuscoOrthoPhylo). Specific tree building implementation with 100 bootstraps on 100% (3117) of protein FASTA files similar between the 14 genomes and was used to create the phylogenomic tree (Supplemental Fig. S3).

### RT-qPCR validation of differentially expressed transcripts

Real Time quantitative PCR (RT-qPCR) was used with gene specific primers to confirm gene expression of 9 loci which were significantly differentially expressed between isolates that included two housekeeping genes. A list of primers designed and used in this study are included in additional file 5. Amplicon sizes for P11 ranged from 81 to 199 bp and 103 to 189 by for G10. Both the acetone and difenoconazole treatments were assessed for log_2_fold change compared to housekeeping genes. Briefly, the RT-qPCR method involved reverse transcription of one micro gram of total RNA from the exact same samples used for the RNA-seq experiment to make cDNA using the iScript cDNA synthesis kit (Bio-Rad). Synthesized cDNA was used as template with the Promega qPCR master mix with a final volume of 10 μl which included 1 μl of cDNA as template, 8ul of master mix, and 0.5 μl of gene specific forward primer and 0.5 μl of reverse primer at 10 μM concentration. Cycling parameters were 1 cycle of 95 °C for 3 min followed by 39 cycles of 95 °C for 10 s, 60 °C for 30 s, then 1 cycle of 95 °C for 10 s. Melt curve was conducted at 65 °C to 95 °C for 5 s at 0.5 °C increments on a C1000 Touch thermal cycler CFX96 Real Time PCR machine (Bio-Rad). Two blank no template controls were included in each 96 well plate alongside two housekeeping genes as positive controls for each locus tested for all 16 samples. Normalized expression of the CYP51 genes as compared to the calmodulin gene is expressed as log_2_ (fold change of difenoconazole over acetone) means and standard errors for each isolate.

### Efflux pump inhibition assay

Broad spectrum efflux pump inhibitors, milbemycin oxime and clorgyline, were dissolved in DMSO to make concentrated stocks (25 mg/ml). Conidial suspensions of G10 and P11 were harvested from PDA plates in 1 ml of sterile water (as indicated above but without Tween 20) and adjusted to 1 × 10^4^ conidia/ml and added to 25 μM and 250 μM clorgyline or 10 μM and 100 μM milbemycin oxime in 1.5 ml Eppendorf tubes. Separate control samples (2) consisted of adding DMSO carrier at the maximum concentration and water only to conidial suspensions. Samples were incubated at room temperature with gentle rocking for 24 h, and 20 μl of spore solution was pipetted in a 3-point fashion onto 3 (4.5 cm) Petri plates containing PDA amended with acetone (carrier control) or 5 μg/ml difenoconazole. Petri plates were placed in an incubator at 25 °C and colony diameters were measured using a digital micrometer after 6 days. The inhibition assay was conducted twice.

### Statistical analyses

The effect of inhibitor treatments on G10 and P11 isolates grown on acetone and difenoconazole was determined by generalized linear mixed models using the PROC GLIMMIX procedure of SAS (Version 9.4, Cary, NC). Differences between treatments were determined using the LSMEANS procedure in SAS 9.4 at the α = 0.05 level of significance with an adjustment for Tukey’s HSD to control for family-wise error. To help explain relationships between gene expression in fold change determined by RNAseq and qRT-PCR, a linear regression analysis (SAS v9.4; PROC REG) was completed for all conditions for G10 and P11 at the α = 0.05 level of significance.

## Supplementary information

**Additional file 1: Fig. S1.** *Penicillium* spp. isolates growing on diagnostic media showing A. the top and B. bottom of the plates.

**Additional file 2: Fig. S2.** Growth rates of *Penicillium* spp. isolates on different diagnostic media.

**Additional file 3: Fig. S3.** Whole genome phylogeny of *Penicillium* species isolates.

**Additional file 4: Table S1.** *Penicillium* spp. genome statistics used in phylogenomic tree construction.

**Additional file 5:.** Primers S5. Primers used in this study.

**Additional file 6: Table S2.** Transcriptome statistics for each sample observed across all 16 samples.

## Data Availability

All data analyzed during this study are included in this published article and its additional information files. The raw data are available freely online as listed below. RNA-seq raw and processed data: To review GEO accession GSE145355: Go to https://www.ncbi.nlm.nih.gov/geo/query/acc.cgi?acc=GSE145355 NCBI Genbank genome submission. P11 genome: BioProject: PRJNA606591: SAMN14098691 BioSample Organism: Penicillium expansum. G10 genome: BioProject: PRJNA606544 BioSample: SAMN14096568 Organism: Penicillium crustosum. Genomes available in NCBI Genbank used in phylogenomic analysis: GCA_000769745.1, GCA_004302965.1, GCA_000002655.1, GCA_000710275.1, GCA_002072265.1, GCA_001307865.1, GCA_002116305.1, GCA_000737485.2, GCA_000769735.1, GCA_002072235.1, GCA_000970515.2, GCA_000577495.1.

## Abbreviations

KEGG: Kyoto Encyclopedia of Genes and Genomes
PDA: Potato dextrose agar
MFS: Major facilitator superfamily
ABC: ATP-binding cassette
PDR: Pleiotropic drug resistance
