# Supplementary material for: Global transcriptomic responses orchestrate difenoconazole resistance in Penicillium spp. causing blue mold of stored apple fruit
Source: BMC Genomics. 2020 Aug 24;21:574. doi: 10.1186/s12864-020-06987-z (PMC7444271; doi:10.1186/s12864-020-06987-z)

**Supplemental Figure 1**. *Penicillium* spp. isolates growing on various diagnostic media. A) Top view of *Penicillium* spp. cultures (G10 *Penicillium crustosum* and P11 *P. expansum*) from this study and two reference isolates from USDA-ARS (NRRL 976 *P. expansum* type strain and NRRL 968 *P. crustosum*) growing on Czapek Yeast Agar (CYA), Malt Extract Agar (MEA), Yeast Extract with Supplements (YES) at 25°C for 7 days. B. Bottom views of *Penicillium* spp. cultures growing on diagnostic medium as stated above.

A.

 **CYA**

**YES**

**MEA**

**NRRL 976 NRRL 968**

**G10 P11 G10 P11**


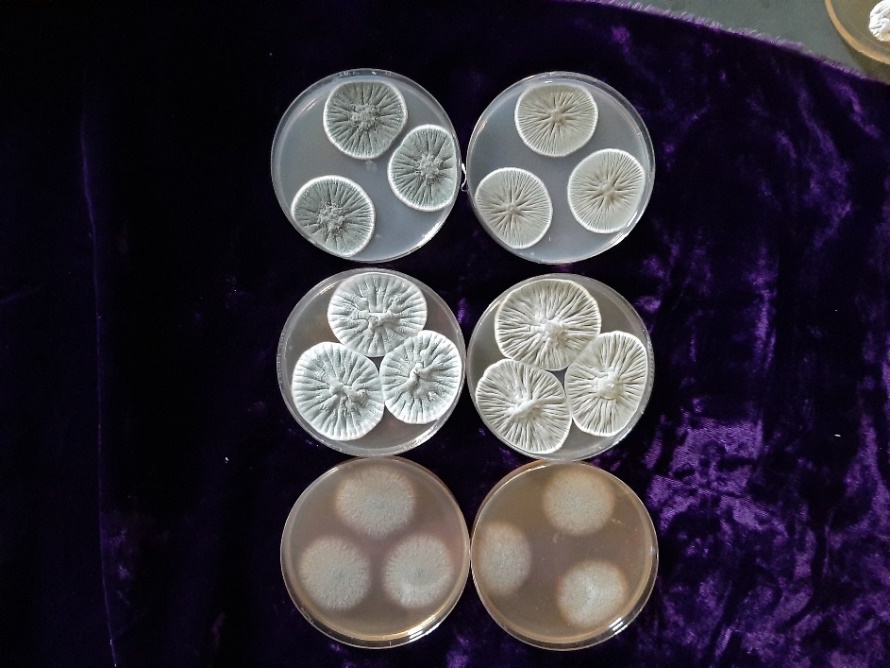


**CYA**

**MEA**

**YES**


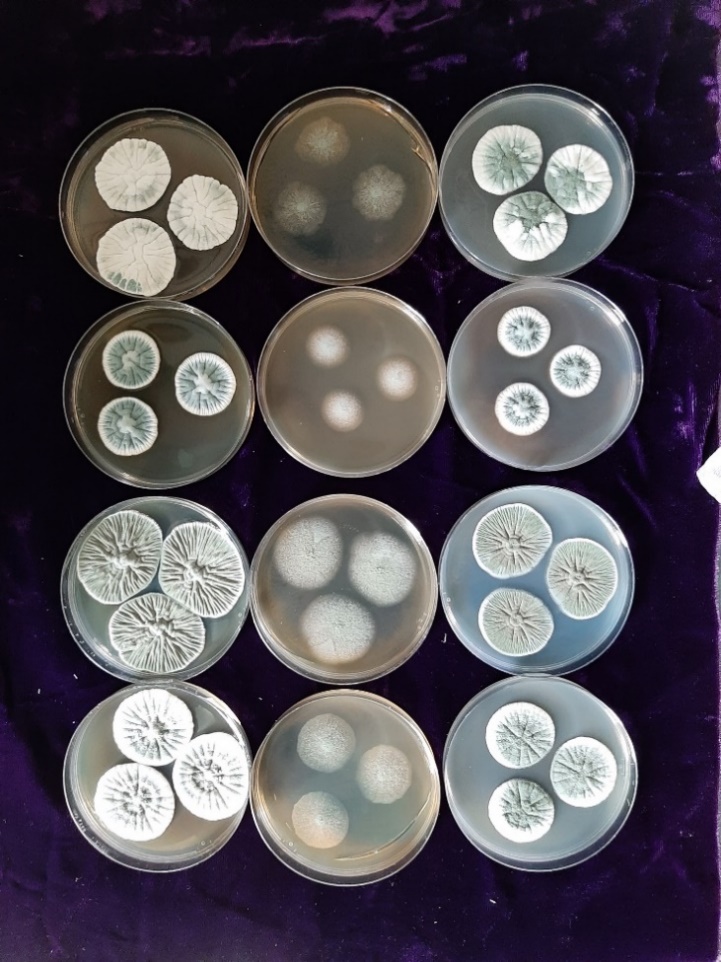


B.

 **CYA**

**YES**

**MEA**

**CYA**

**YES**

**MEA**


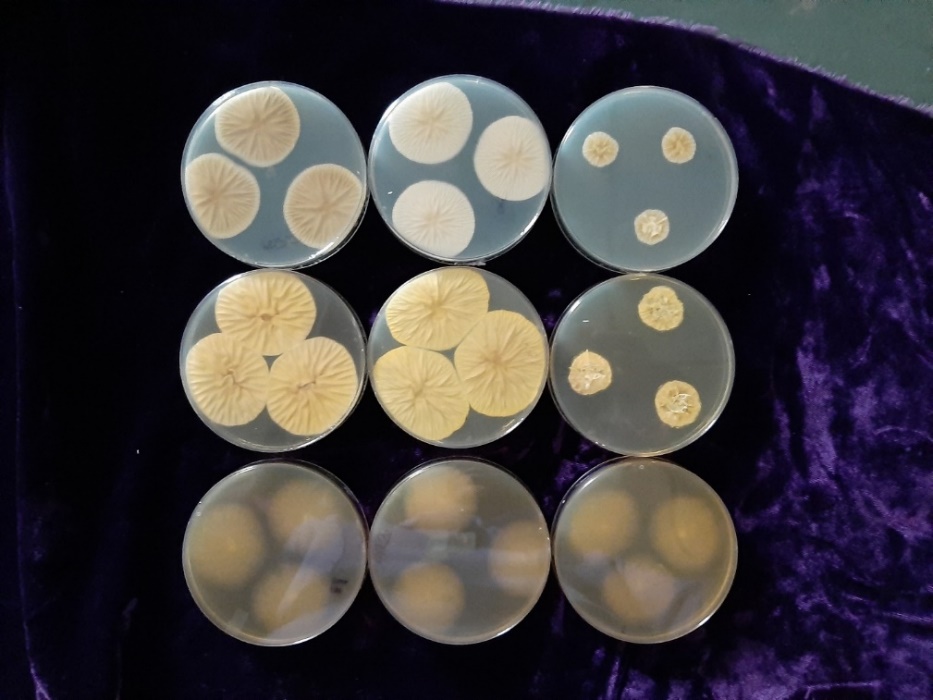


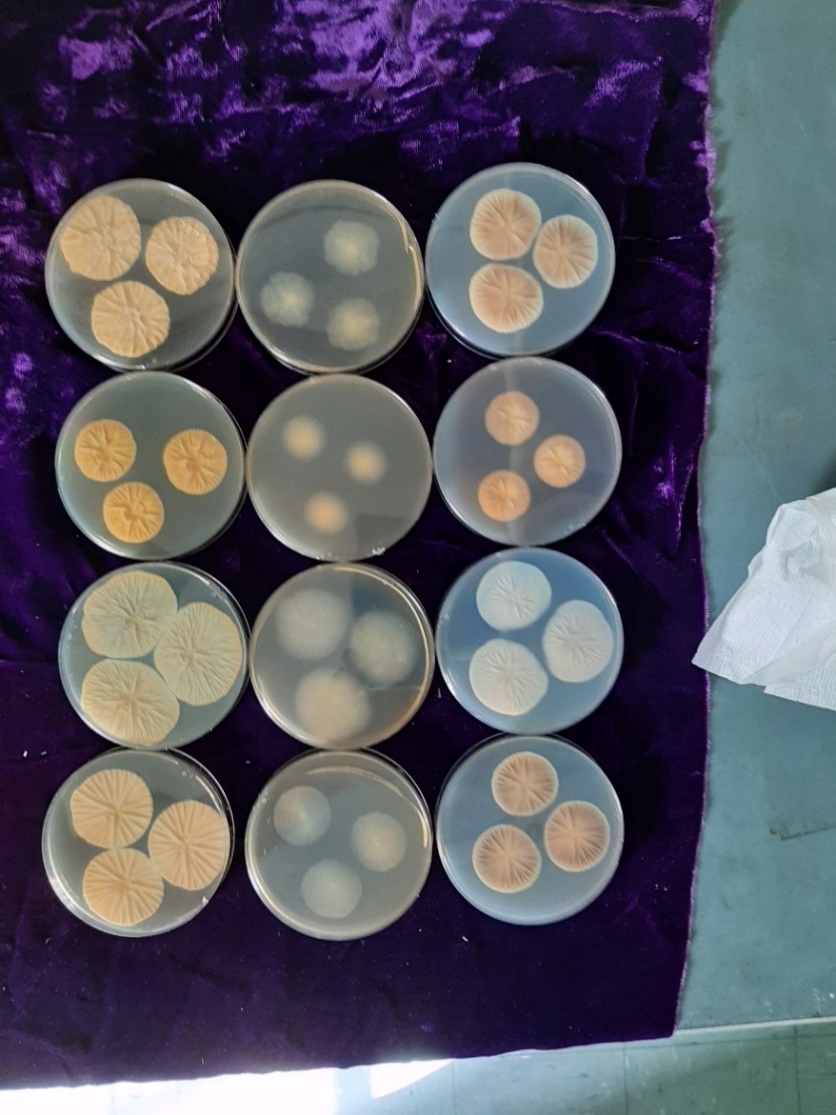

Supplement: Supplementary file 1 — Additional file 1: Fig. S1. Penicillium spp. isolates growing on diagnostic media showing A. the top and B. bottom of the plates. [file 12864_2020_6987_MOESM1_ESM.docx]
