# Supplementary figures and images for: Global transcriptomic responses orchestrate difenoconazole resistance in Penicillium spp. causing blue mold of stored apple fruit
Source: BMC Genomics. 2020 Aug 24;21:574. doi: 10.1186/s12864-020-06987-z (PMC7444271; doi:10.1186/s12864-020-06987-z)

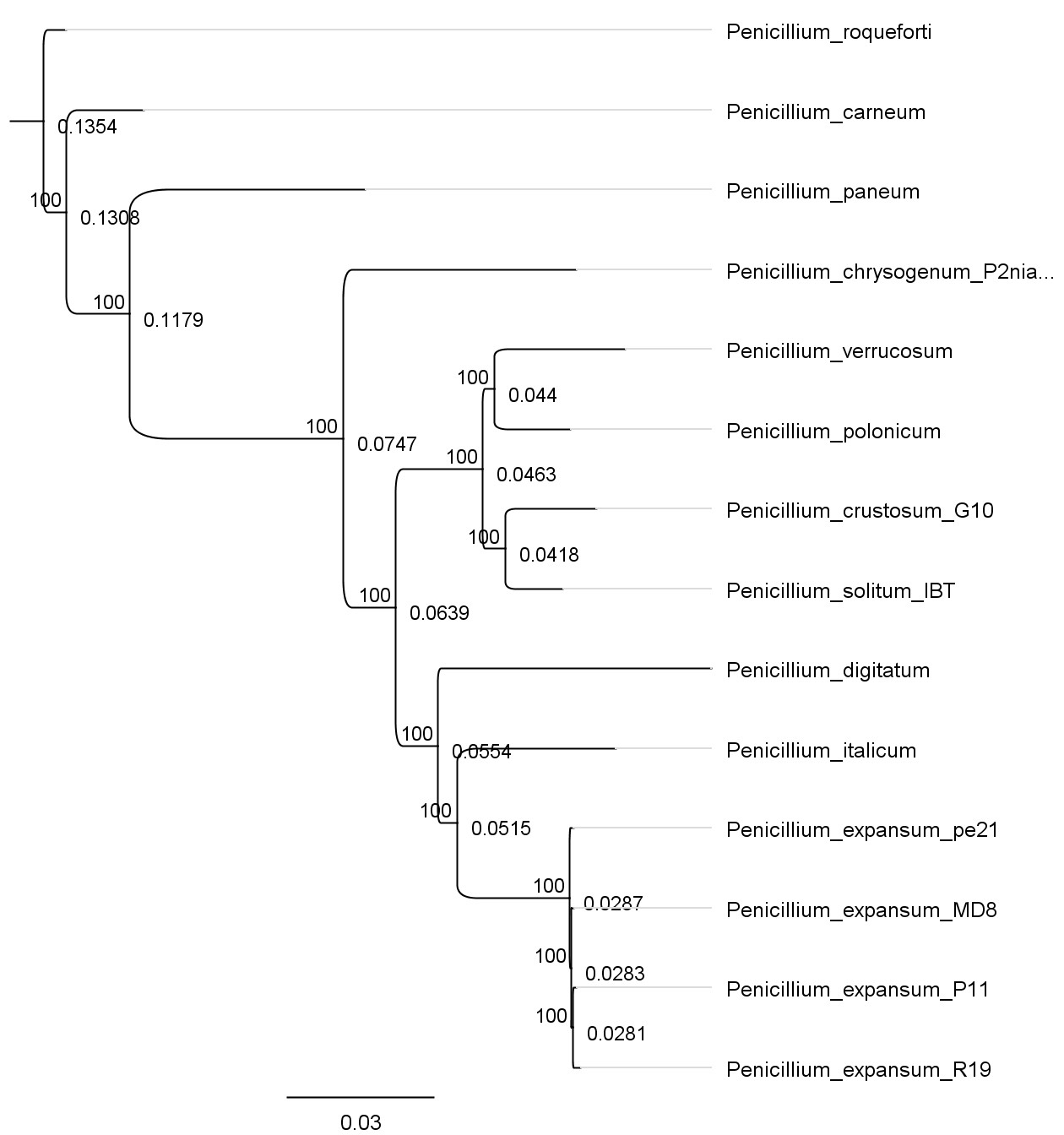

Supplement: Supplementary file 3 — Additional file 3: Fig. S3. Whole genome phylogeny of Penicillium species isolates. [file 12864_2020_6987_MOESM3_ESM.jpg]
