# Supplementary material for: Global transcriptomic responses orchestrate difenoconazole resistance in Penicillium spp. causing blue mold of stored apple fruit
Source: BMC Genomics. 2020 Aug 24;21:574. doi: 10.1186/s12864-020-06987-z (PMC7444271; doi:10.1186/s12864-020-06987-z)
Supplement: Supplementary file 5 — Additional file 5:. Primers S5. Primers used in this study. [file 12864_2020_6987_MOESM5_ESM.docx]

Supplemental File S6. Primers used in this study.

For cDNA P11

CYP51-P-F GACAACGTCAACGGCAGGGA

CYP51-P-R AATTGCGGACGACGAGTGCT

ERG6-P-F GAGATTCGTGAGGCCCGCAA

ERG6-P-R TAGGCGAAGCGGCAGAAGTG

GLYCOHYDRO-P-F CCCAACAGCAACGTCCAGGT

GLYCOHYDRO-P-F GGCGACAGTGGTGGTGGAAA

MFS-P-F **TACAAGCGAGAGGAACAA**

MFS-P-R **CAATAGACCACCGACAATC**

PATC-P-F CTCCCGCTGGGCAAAGCATA

PATC-P-R CTAGGAGCAGCACCGCAGAG

PacC-P-F ACACCTCCAACCGCTCTCCT

PacC-P-R GTGGTCATACGCACCGCTCA

PATL-P-F AGCCAGCTCTCGATGACCCT

PATL-P-R TCGTGAGGAGGACTGTGCCA

PEPG1-P-F GACTGGTCTGGCCTCGGGTA

PEPG1-P-R TGAGGTGACCGGAAGCTCCA

CYP52-P-F **GGATATGTCTAGGCAAGGA**

CYP52-P-R **CTCATAACCACAATCGTCAA**

RPB2-P-F **GAGCAGATGAAGGAGGAA**

RPB2-P-R **TAACCACCAGAGTCGTAAG**

For cDNA G10

CYP51-G-F GGGCACCACTGCCTCACAAT

CYP51-G-R CCCTGCCGTTGACGTTGTCT

ERG6-G-F GAGGCCCGCAAGGCTGAATA

ERG6-G-F TAGGCGAAGCGGCAGAAGTG

GLYCOHYDRO-G-F CCTGACCGACTCCGAGGACA

GLYCOHYDRO-G-R ACCGACTGGAGACGCTCCTT

MFS-G-F **TGGCTTGCTATGCTTGTA**

**MFS-G-R TTATCCTTCTTGGCGTTCT**

PATC-G-F **GAGCAGCCTGTGTATGTT**

PATC-G-R **CAGATTGAGATAGAATGACCATC**

PACC-G-F **CCGCTCTCCTATCTCCAT**

**PACC-G-R GTCATACGCACCACTCAG**

PATL-G-F **AAGCCATCAGCCTTAGTC**

PATL-G-R **ATCCAGCATCGGAATCTC**

PEPG1-G-F **CCAACAGCAAGACCAATC**

PEPG1-G-R **AGAGAATCCTTATCGCCATT**

CYP52-G-F **GAGCAGAGACGGATACTT**

CYP52-G-R **GCATATTCCTTGACGACAT**

RPB2-G-F **CGGTGGATTGAAGTATGC**

RPB2-G-R **GAGGAAGCGAATGTGTAAC**
