# Supplementary material for: Global transcriptomic responses orchestrate difenoconazole resistance in Penicillium spp. causing blue mold of stored apple fruit
Source: BMC Genomics. 2020 Aug 24;21:574. doi: 10.1186/s12864-020-06987-z (PMC7444271; doi:10.1186/s12864-020-06987-z)
Supplement: Supplementary file 5 [file 12864_2020_6987_MOESM2_ESM.docx]

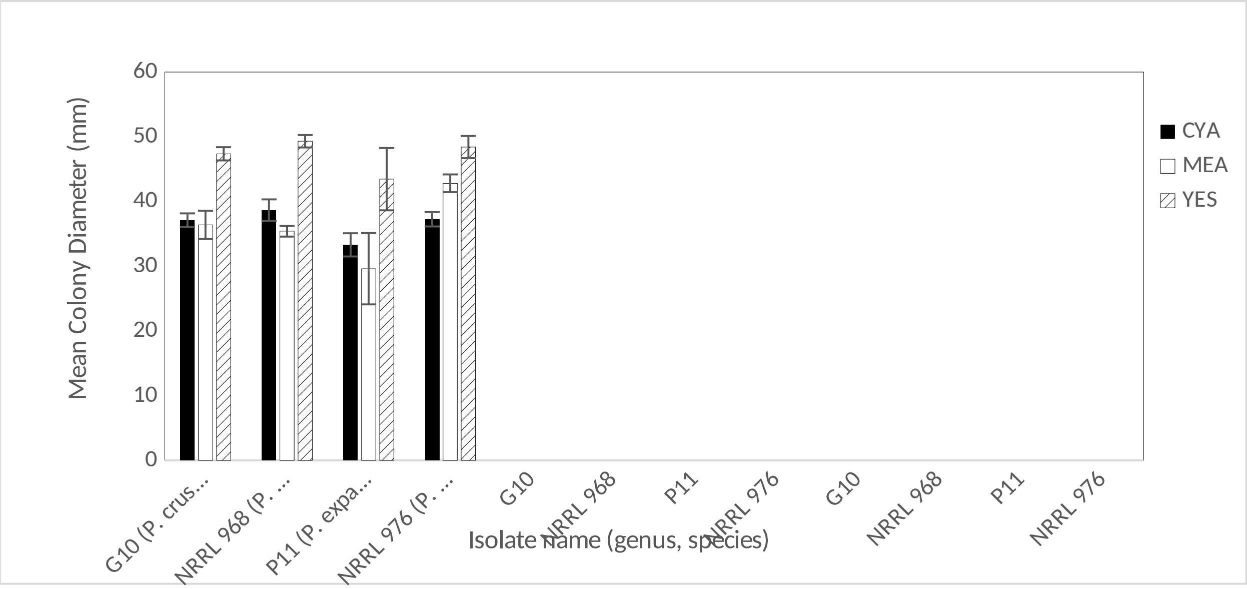
Mean Colony Diameter (mm)
60
50
40
30
20
10
0
G10 (P. crus…
NRRL 968 (P. …
P11 (P. expa…
NRRL 976 (P. …
G10
NRRL 968
P11
NRRL 976
G10
NRRL 968
P11
NRRL 976
Isolate name (genus, species)
CYA
MEA
YES
